# Earlobe Loss After Herpes Zoster Infection: An Uncommon Complication

**DOI:** 10.1111/jocd.16642

**Published:** 2024-10-25

**Authors:** Anissa Zaouak, Amal Chamli, Houda Hammami, Samy Fenniche

**Affiliations:** ^1^ Faculty of Medicine of Tunis University of Tunis El Manar Tunis Tunisia; ^2^ Dermatology Department Habib Thameur Hospital Tunis Tunisia

Dear sir,

A 57‐year‐old female with a significant past medical history of Hodgkin's lymphoma, which had been treated with both chemotherapy and radiation therapy, presented to our dermatology department with an acute onset of a painful vesicular rash. The rash had been present for 4 days and was localized to her face and neck (Figure 1). Upon clinical examination, the rash was found to be distributed along the right mandibular division of the trigeminal nerve, extending to the neck and involving the right ear on the same side. Despite the extensive nature of the rash, there were no accompanying neurological deficits such as facial paralysis or hearing loss.

**FIGURE 1:**
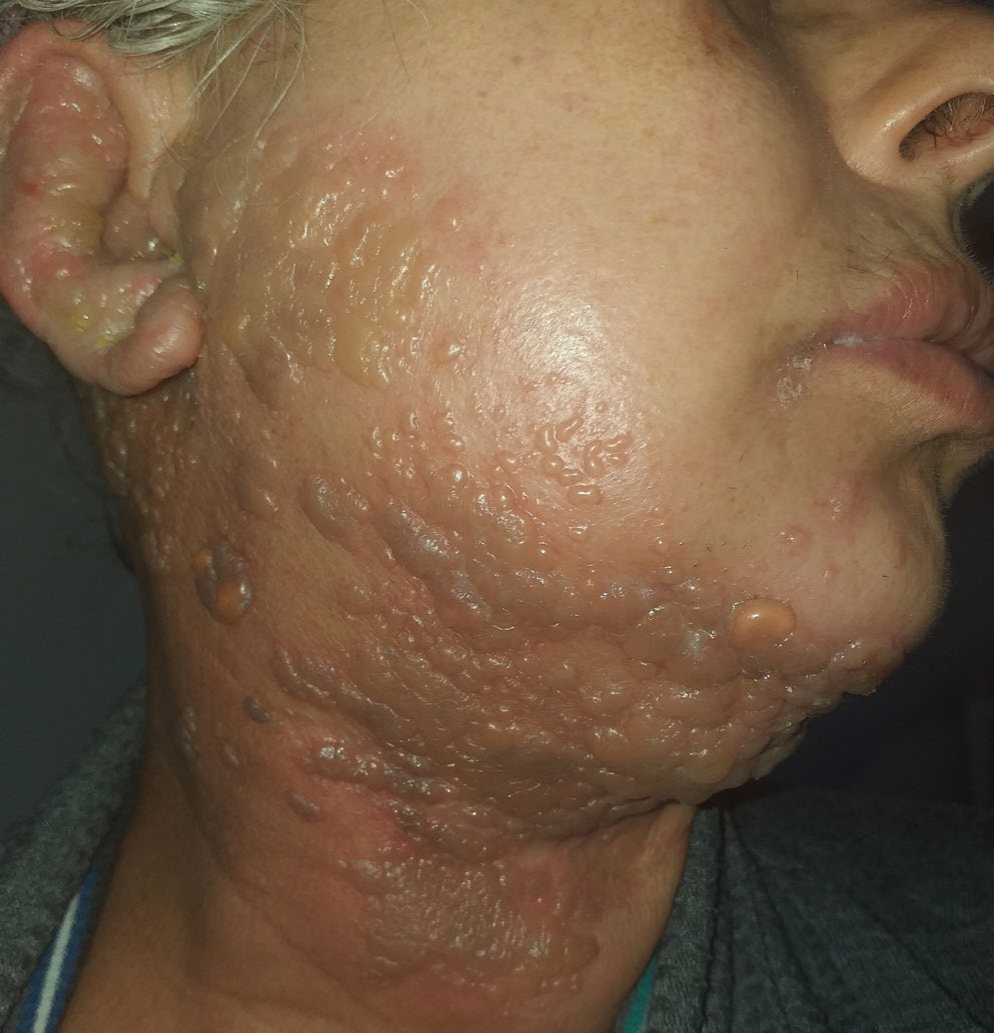
Vesicular rash affecting the right mandibular division of trigeminal nerve, the neck, and the homolateral ear.

Given the severity of her symptoms and her immunocompromised status, the patient was promptly admitted to the hospital. She was initiated on intravenous acyclovir at a dosage of 10 mg/kg/day to target the underlying viral infection. Additionally, pain management was provided with paracetamol, and supportive care measures were implemented. After 10 days of intensive medical treatment, the patient showed significant improvement, with a complete resolution of the vesicular lesions and pain.

One month after the initial presentation, a follow‐up examination revealed residual post‐inflammatory hyperpigmentation on the neck and persistent edema of the right ear, with a noticeable retraction of the right earlobe. Over the following months, the patient continued to be monitored closely. At the five‐month follow‐up, it was observed that the right earlobe had completely disappeared (Figure 2), a rare and unusual complication following herpes zoster.

**FIGURE 2:**
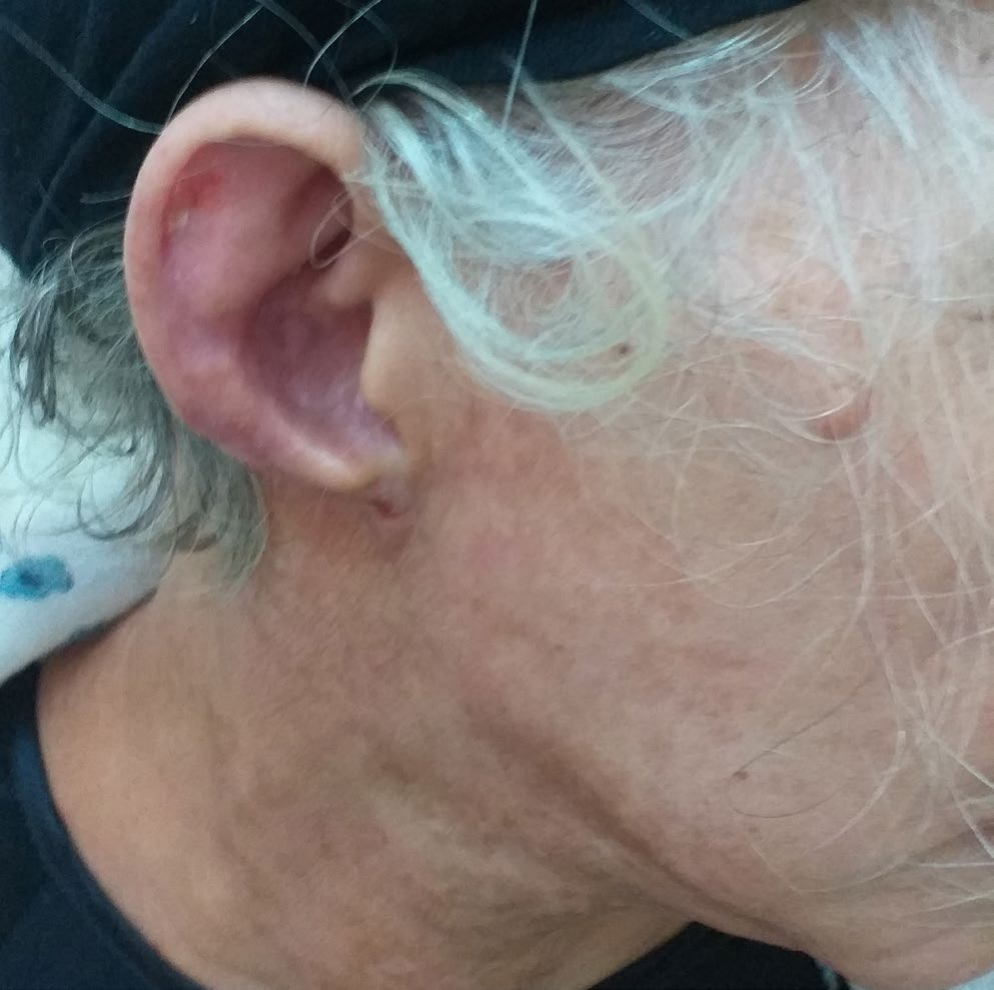
Clinical follow‐up after 5 months of the episode revealed a total disappearance of the right earlobe.

Herpes zoster (HZ), caused by the reactivation of the varicella–zoster virus (VZV), is a common skin infection, particularly in elderly or immunocompromised individuals. While HZ can result in various complications, including postherpetic neuralgia, bacterial superinfection, aseptic meningitis, and even visceral involvement, earlobe loss is an unprecedented finding in the medical literature [1]. The early initiation of antiviral therapy and appropriate pain management in this case likely mitigated the risk of postherpetic neuralgia; yet, other complications, such as nonspecific granulomatous dermatitis, granulomatous vasculitis, pseudolymphoma, and keloid formation, can still occur [1, 2, 3].

The pathophysiology behind the earlobe loss in this case remains unclear. However, it is hypothesized that the severity of the initial herpes zoster infection, coupled with the extensive involvement of the hemiface, neck, and ear, may have contributed to this rare outcome, particularly given the patient's immunocompromised state.

In our patient, the clinical presentation was typical of herpes zoster. However, when the clinical diagnosis is not clear, we should take a cutaneous swab from the vesicular lesions and perform a PCR to detect varicella–zoster virus's DNA to confirm the diagnosis.

A review of the literature indicates that cutaneous complications following herpes zoster can manifest either immediately after the acute vesicular eruption has resolved or even several weeks later. These complications are often attributed to type III or type IV hypersensitivity reactions, and the phenomenon known as Koebner's phenomenon has also been suggested as a potential trigger [1, 2]. Notably, the VZV genome is typically not detectable by PCR in these late post‐zoster cutaneous reactions [2].

Currently, herpes zoster is a vaccine‐preventable disease. Healthcare professionals, particularly oncologists, should be vigilant about vaccinating immunocompromised patients undergoing chemotherapy. Ideally, vaccination should occur 2–3 weeks prior to the start of oncological treatments. For these vulnerable patients, the recombinant vaccine is recommended to prevent herpes zoster and its associated complications [4, 5].

Therefore, in immunocompromised patients who experience a severe case of herpes zoster, prolonged clinical follow‐up is crucial to monitor for and manage any late‐onset cutaneous complications that may arise.

## Author Contributions

Anissa Zaouak and Amal Chamli wrote the manuscript with support from Houda Hammami. Anissa Zaouak analyzed the data. Samy Fenniche supervised the project. All authors have read and approved the final manuscript.

## Ethics Statement

The authors certify that they have obtained all appropriate patient consent forms, in which the patients gave their consent for images and other clinical information to be included in the journal.

## Conflicts of Interest

The authors declare no conflicts of interest.

## Data Availability

The data that support the findings of this study are available on request from the corresponding author. The data are not publicly available due to privacy or ethical restrictions.
